# Supplementary material for: Efficacy and Safety of TiNO-Coated Stents versus Drug-Eluting Stents in Acute Coronary Syndrome: Systematic Literature Review and Meta-Analysis
Source: Biomedicines. 2022 Dec 7;10(12):3159. doi: 10.3390/biomedicines10123159 (PMC9775300; doi:10.3390/biomedicines10123159)
Supplement: Supplementary file 1 [file biomedicines-10-03159-s001.zip › biomedicines-1986514-supplementary.pdf]

Efficacy and safety of TiNO-coated stents versus drug-eluting stents in acute coronary syndrome. Systematic literature review and meta-analysis.

SUPPLMENTARY MATERIALS

Frederic C. Daoud, Louis Létinier , Nicholas Moore, Pierre Coste and Pasi P. Karjalainen

ADDITIONAL FOREST PLOTS OF POOLED RISK RATIOS:

Key results of all plots and sensitivity analysis are reported in the article , Table 2.

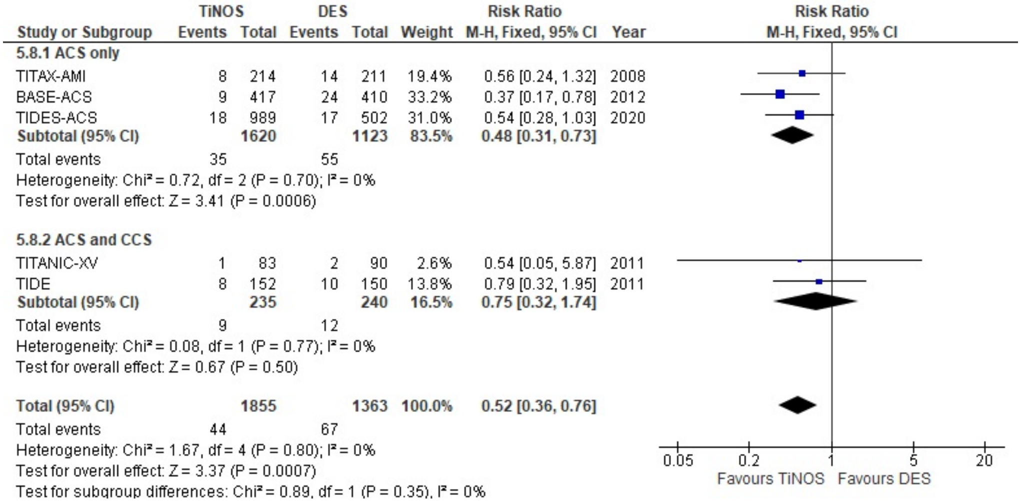

Figure S1. Recurrent non-fatal MI – 1 year.

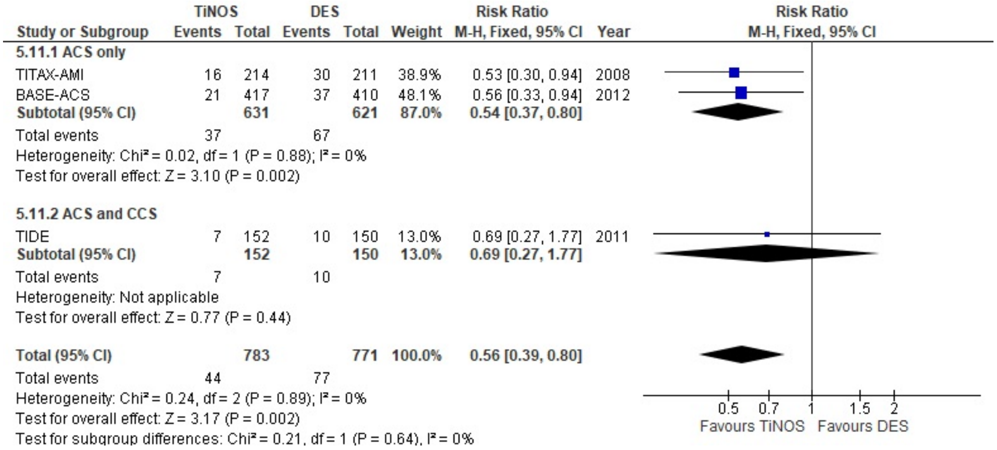

Figure S2. Recurrent non-fatal MI – 5 years.

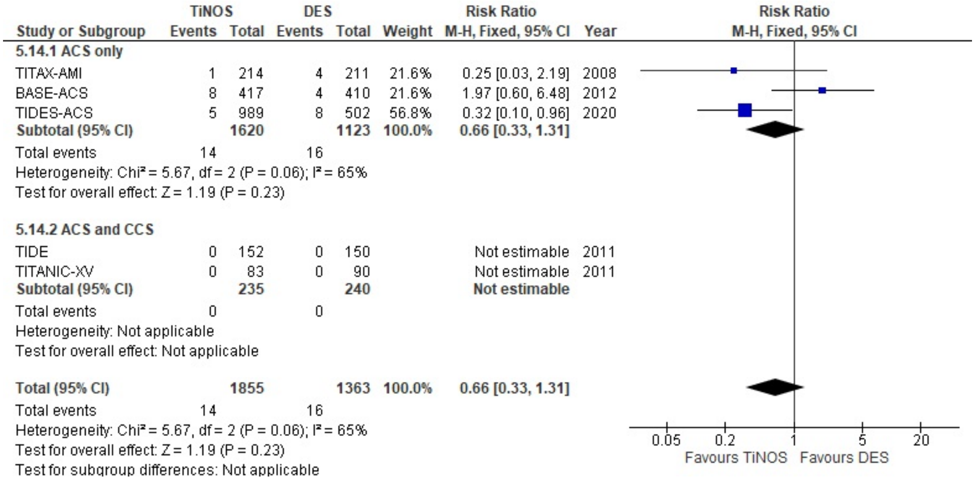

Figure S3. CD – 1 year.

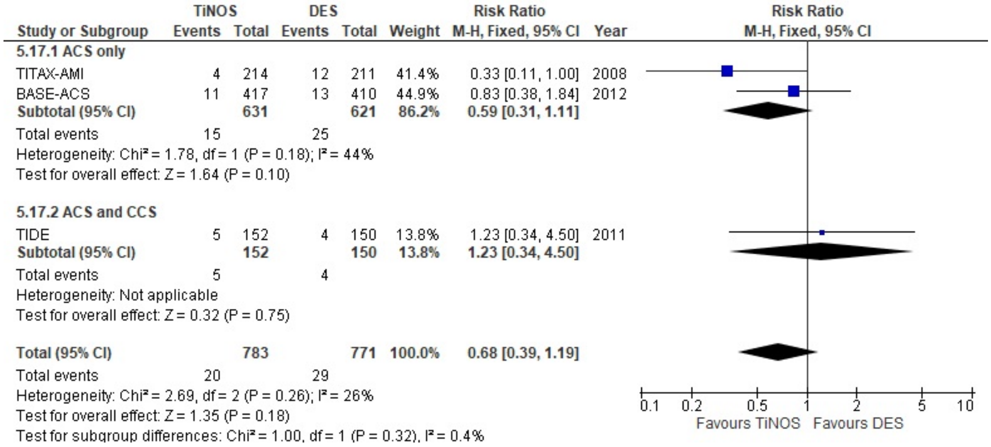

Figure S4. CD – 5 year.

Study or Subgroup	TiNOS Events	TiNOS Total	DES Events	DES Total	Weight	Risk Ratio M-H, Fixed, 95% CI	Year
**5.11.1 ACS only**							
TITAX-AMI	16	214	30	211	38.9%	0.53 [0.30, 0.94]	2008
BASE-ACS	21	417	37	410	48.1%	0.56 [0.33, 0.94]	2012
**Subtotal (95% CI)**		**631**		**621**	**87.0%**	**0.54 [0.37, 0.80]**	
Total events	37		67				
Heterogeneity: Chi² = 0.02, df = 1 (P = 0.88); I² = 0%							
Test for overall effect: Z = 3.10 (P = 0.002)							
**5.11.2 ACS and CCS**							
TIDE	7	152	10	150	13.0%	0.69 [0.27, 1.77]	2011
**Subtotal (95% CI)**		**152**		**150**	**13.0%**	**0.69 [0.27, 1.77]**	
Total events	7		10				
Heterogeneity: Not applicable							
Test for overall effect: Z = 0.77 (P = 0.44)							
**Total (95% CI)**		**783**		**771**	**100.0%**	**0.56 [0.39, 0.80]**	
Total events	44		77				
Heterogeneity: Chi² = 0.24, df = 2 (P = 0.89); I² = 0%							
Test for overall effect: Z = 3.17 (P = 0.002)							
Test for subgroup differences: Chi² = 0.21, df = 1 (P = 0.64), I² = 0%							

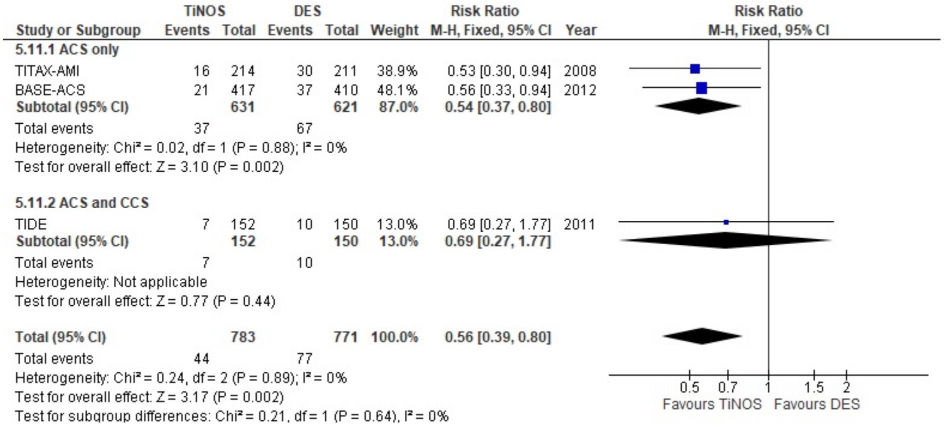

Figure S5. Probable or definite ST – 1 year.

Study or Subgroup	TiNOS Events	TiNOS Total	DES Events	DES Total	Weight	Risk Ratio M-H, Fixed, 95% CI	Year
**5.29.1 ACS only**							
TITAX-AMI	2	214	15	211	47.6%	0.13 [0.03, 0.57]	2008
BASE-ACS	6	417	16	410	50.8%	0.37 [0.15, 0.93]	2012
**Subtotal (95% CI)**		**631**		**621**	**98.4%**	**0.25 [0.12, 0.55]**	
Total events	8		31				
Heterogeneity: Chi² = 1.40, df = 1 (P = 0.24); I² = 28%							
Test for overall effect: Z = 3.49 (P = 0.0005)							
**5.29.2 ACS and CCS**							
TIDE	1	152	0	150	1.6%	2.96 [0.12, 72.11]	2011
**Subtotal (95% CI)**		**152**		**150**	**1.6%**	**2.96 [0.12, 72.11]**	
Total events	1		0				
Heterogeneity: Not applicable							
Test for overall effect: Z = 0.67 (P = 0.51)							
**Total (95% CI)**		**783**		**771**	**100.0%**	**0.30 [0.14, 0.61]**	
Total events	9		31				
Heterogeneity: Chi² = 3.39, df = 2 (P = 0.18); I² = 41%							
Test for overall effect: Z = 3.31 (P = 0.0009)							
Test for subgroup differences: Chi² = 2.15, df = 1 (P = 0.14), I² = 53.4%							

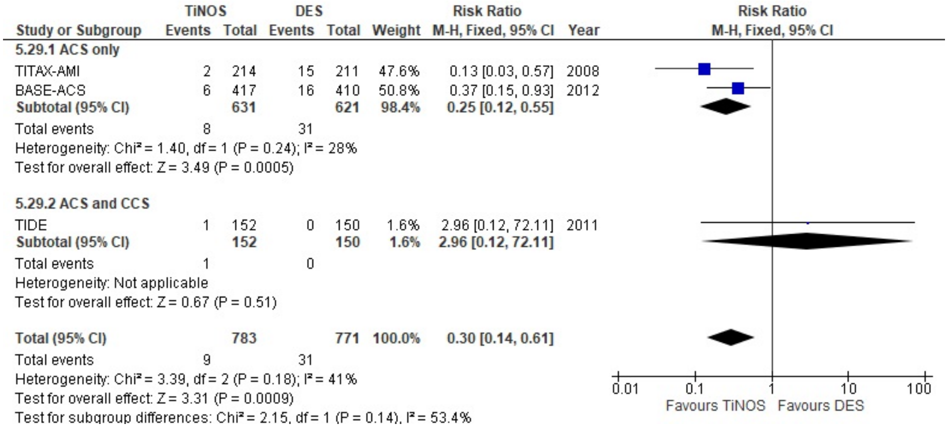

Figure S6. Probable or definite ST – 5 year.

INELIGIBLE RECORDS:

n = 58 among 67 non-duplicates. Did not meet the review's PICOS eligibility criteria.

1. Sia, J.; Nammas, W.; Collet, C.; De Bruyne, B.; Karjalainen, P. Comparative study of neointimal coverage between titanium-nitric oxide-coated and everolimus-eluting stents in acute coronary syndromes ORIGINAL (NON-ENGLISH) TITLE Estudio comparativo de la cobertura neointimal entre los stents con recubrimiento de titanio-óxido nítrico y los liberadores de everolimus en el sindrome coronario agudo. *Revista Espanola de Cardiologia (2022). Date of Publication: 2022*, doi:10.1016/j.recesp.2022.05.011.
2. Grube, E.; Buellesfeld, L. BioMatrix Biolimus A9-eluting coronary stent: a next-generation drug-eluting stent for coronary artery disease. *Expert review of medical devices* **2006**, *3*, 731-741, doi:10.1586/17434440.3.6.731.
3. Wu, P.; Grainger, D.W. Drug/device combinations for local drug therapies and infection prophylaxis. *Biomaterials* **2006**, *27*, 2450-2467, doi:10.1016/j.biomaterials.2005.11.031.
4. Coolong, A.; Kuntz, R.E. Understanding the drug-eluting stent trials. *The American journal of cardiology* **2007**, *100*, 17k-24k, doi:10.1016/j.amjcard.2007.06.004.
5. Konorza, T.F.M.; Author, A.; Correspondence, A.; T.F.M. Konorza, E.G.; Full Record Entry, D. Prospective, multi-center randomized trial to compare the implantation of a titanium-nitride-oxide coated stent with a paclitaxel stent in patients with acute myocardial infarction ORIGINAL (NON-ENGLISH) TITLE Prospektive, randomisierte, multizentrische studie zum vergleich der ergebnisse eines titanium-nitritoxid-beschichteten stents mit einem paclitaxel freisetzenden stent bei patienten mit akutem myokardinfarkt. *Herz* **2007**, *32*, 513, doi:10.1007/s00059-007-3036-6.
6. van der Hoeven, B.; Liem, S.; Jukema, J.; Suraphakdee, N.; Putter, H.; Dijkstra, J.; Atsma, D.; Bootsma, M.; Zeppenfeld, K.; Oemrawsingh, P.; et al. Sirolimus-Eluting Stents Versus Bare-Metal Stents in Patients With. *Journal of the American College of Cardiology* **2008**, *51*, 618-626, doi:10.1016/j.jacc.2007.09.056.
7. van der Hoeven, B.; Liem, S.; Dijkstra, J.; Bergheanu, S.; Putter, H.; Antoni, M.; Atsma, D.; Bootsma, M.; Zeppenfeld, K.; Jukema, J.; et al. Stent Malapposition After Sirolimus-Eluting and Bare-Metal Stent Implantation in Patients with ST-Segment Elevation Myocardial Infarction. Acute and 9-Month Intravascular Ultrasound Results of the MISSION! Intervention Study. *JACC: Cardiovascular Interventions* **2008**, *1*, 192-201, doi:10.1016/j.jcin.2008.02.003.
8. Atary, J.; Bergheanu, S.; Van Der Hoeven, B.; Atsma, D.; Bootsma, M.; Van Der Kley, F.; Zeppenfeld, K.; Jukema, J.; Schalij, M. Impact of sirolimus-eluting stent implantation compared to bare-metal stent implantation for acute myocardial infarction on coronary plaque composition at nine months follow-up: A Virtual Histology intravascular ultrasound analysis. Results from the Leiden MISSION! Intervention study. *EuroIntervention : journal of EuroPCR in collaboration with the Working Group on Interventional Cardiology of the European Society of Cardiology* **2009**, *5*, 565-572, doi:10.4244/eijv5i5a92.
9. de Luca, G.; Valgimigli, M.; Spaulding, C.; Menichelli, M.; Brunner-La Rocca, H.; van der Hoeven, B.; Di Lorenzo, E.; de la Llera, L.; Pasceri, V.; Pittl, U.; et al. Short and long-term benefits of sirolimus-eluting stent in ST-segment elevation myocardial infarction: A meta-analysis of randomized trials. *Journal of Thrombosis and Thrombolysis* **2009**, *28*, 200-210, doi:10.1007/s11239-009-0305-7.
10. Karjalainen, P.; Ylitalo, A.; Niemelä, M.; Kervinen, K.; Mäkikallio, T.; Pietilä, M.; Sia, J.; Tuomainen, P.; Nyman, K.; Airaksinen, K. Two-year follow-up after percutaneous coronary intervention with titanium-nitride-oxide-coated stents versus paclitaxel-eluting stents in

acute myocardial infarction. *Annals of medicine* **2009**, *41*, 599-607, doi:10.1080/07853890903111018.

11. Sant'Anna, F.; Batista, L.; Brito, M.; Menezes, S.; Ventura, F.; Buczynski, L.; Barrozo, C. Randomized comparison of percutaneous coronary intervention with titanium-nitride-oxide-coated stents versus stainless steel stents in patients with coronary artery disease: RIO trial ORIGINAL (NON-ENGLISH) TITLE Estudo randomizado e comparativo da intervenção coronária percutânea com stents recobertos por titânio- óxido nítrico ou de aço inoxidável em pacientes com doença arterial coronária: Estudo RIO. *Revista Brasileira de Cardiologia Invasiva* **2009**, *17*, 69-75.
12. Atary, J.Z.; van der Hoeven, B.L.; Liem, S.S.; Jukema, J.W.; van der Bom, J.G.; Atsma, D.E.; Bootsma, M.; Zeppenfeld, K.; van der Wall, E.E.; Schalij, M.J. Three-Year Outcome of Sirolimus-Eluting Versus Bare-Metal Stents for the Treatment of ST-Segment Elevation Myocardial Infarction (from the MISSION! Intervention Study). *Am. J. Cardiol.* **2010**, *106*, 4-12, doi:10.1016/j.amjcard.2010.02.005.
13. Dibra, A.; Tiroch, K.; Schulz, S.; Kelbæk, H.; Spaulding, C.; Laarman, G.; Valgimigli, M.; Di Lorenzo, E.; Kaiser, C.; Tierala, I.; et al. Drug-eluting stents in acute myocardial infarction: Updated meta-analysis of randomized trials. *Clinical Research in Cardiology* **2010**, *99*, 345-357, doi:10.1007/s00392-010-0133-y.
14. Konorza, T.F.M.; Author, A.; Correspondence, A.; T. F. M. Konorza, E.G.; Ai, P.I.E.D.; Full Record Entry, D. Randomized comparison of titanium-nitride-oxide coated stents with zotarolimus-eluting stents for coronary revascularisation ORIGINAL (NON-ENGLISH) TITLE Prospektive randomisierte studie zum vergleich des titan-nitrid- beschichteten stents mit dem zotarolimus-freisetzenden stent zur koronarrevaskularisation. *Herz* **2010**, *35*, 364, doi:10.1007/s00059-010-3359-6.
15. Morice, M.; Van Hout, B.; Berland, J.; Bessou, J.; Carrié, D.; Dawkins, K.; Mohr, F.; Serruys, P. Economic evaluation of the French subset of the SYNTAX trial at 1 year: Drug-eluting stents compared with bypass surgery for patients with 3-vessel and/or left main coronary artery disease. *Archives of Cardiovascular Diseases Supplements* **2010**, *2*, 20, doi:10.1016/s1878-6480(10)70061-6.
16. Moschovitis, A.; Simon, R.; Seidenstucker, A.; Klauss, V.; Baylacher, M.; Luscher, T.F.; Moccetti, T.; Windecker, S.; Meier, B.; Hess, O.M. Randomised comparison of titanium-nitride-oxide coated stents with bare metal stents: five year follow-up of the TiNOX trial. *EuroIntervention : journal of EuroPCR in collaboration with the Working Group on Interventional Cardiology of the European Society of Cardiology* **2010**, *6*, 63-68, doi:10.4244/EIJV6I1A10.
17. Karjalainen, P.; Nammas, W. Bioactive stents for percutaneous coronary intervention: A new forerunner on the track. *Interventional Cardiology* **2011**, *3*, 527-529, doi:10.2217/ica.11.61.
18. Boden, H.; van der Hoeven, B.L.; Liem, S.S.; Atary, J.Z.; Cannegieter, S.C.; Atsma, D.E.; Bootsma, M.; Jukema, J.W.; Zeppenfeld, K.; Oemrawsingh, P.V.; et al. Five-year clinical follow-up from the MISSION! Intervention Study: sirolimus-eluting stent versus bare metal stent implantation in patients with ST-segment elevation myocardial infarction, a randomised controlled trial. *EuroIntervention : journal of EuroPCR in collaboration with the Working Group on Interventional Cardiology of the European Society of Cardiology* **2012**, *7*, 1021-1029, doi:10.4244/EIJV7I9A164.
19. De Luca, G.; Dirksen, M.; Spaulding, C.; Kelbæk, H.; Schalij, M.; Thuesen, L.; Van Der Hoeven, B.; Vink, M.; Kaiser, C.; Musto, C.; et al. Drug-eluting vs bare-metal stents in primary angioplasty: A pooled patient-level meta-analysis of randomized trials. *Archives of Internal Medicine* **2012**, *172*, 611-621, doi:10.1001/archinternmed.2012.758.
20. Lehtinen, T.; Airaksinen, K.E.; Ylitalo, A.; Karjalainen, P.P. Stent strut coverage of titanium-nitride-oxide coated stent compared to paclitaxel-eluting stent in acute myocardial infarction: TITAX-OCT study. *The international journal of cardiovascular imaging* **2012**, *28*, 1859-1866, doi:10.1007/s10554-012-0032-6.

21. Nct. The Titan Versus Everolimus Intracoronary Stent (Xience V) in Diabetic Patients. *https://clinicaltrials.gov/show/NCT01510509* **2012**.
22. Tuomainen, P.; Ylitalo, A.; Niemelä, M.; Kervinen, K.; Pietilä, M.; Sia, J.; Nyman, K.; Nammas, W.; Airaksinen, K.; Karjalainen, P. Gender-based analysis of the 3-year outcome of bioactive stents versus paclitaxel-eluting stents in patients with acute myocardial infarction: An insight from the TITAX-AMI trial. *Journal of Invasive Cardiology* **2012**, *24*, 104-108.
23. De Luca, G.; Dirksen, M.; Spaulding, C.; Kelbk, H.; Schalij, M.; Thuesen, L.; Van der Hoeven, B.; Vink, M.A.; Kaiser, C.; Musto, C.; et al. Impact of Diabetes on Long-Term Outcome After Primary Angioplasty. *Diabetes Care* **2013**, *36*, 1020-1025, doi:10.2337/dc12-1507.
24. De Luca, G.; Dirksen, M.; Spaulding, C.; Kelbæk, H.; Schalij, M.; Thuesen, L.; van der Hoeven, B.; Vink, M.; Kaiser, C.; Musto, C.; et al. Time course, predictors and clinical implications of stent thrombosis following primary angioplasty: Insights from the DESERT cooperation. *Thrombosis and Haemostasis* **2013**, *110*, 826-833, doi:10.1160/th13-02-0092.
25. De Luca, G.; Dirksen, M.; Spaulding, C.; Kelbæk, H.; Schalij, M.; Thuesen, L.; van der Hoeven, B.; Vink, M.; Kaiser, C.; Musto, C.; et al. Meta-analysis comparing efficacy and safety of first generation drug-eluting stents to bare-metal stents in patients with diabetes mellitus undergoing primary percutaneous coronary intervention. *Am. J. Cardiol.* **2013**, *111*, 1295-1304, doi:10.1016/j.amjcard.2013.01.281.
26. Karjalainen, P. Neointimal coverage and vasodilator response to titanium-nitride-oxide-coated bioactive stents and everolimus-eluting stents in patients with acute coronary syndrome: Insights from the BASE-ACS trial. *International Journal of Cardiovascular Imaging* **2013**, *29*, 1693-1703, doi:10.1007/s10554-013-0285-8.
27. Romppanen, H.; Nammas, W.; Kervinen, K.; Mikkelsson, J.; Pietilä, M.; Lalmand, J.; Rivero-Crespo, F.; Pentikäinen, M.; Tedjokusumo, P.; Karjalainen, P.P. Outcome of ST-elevation myocardial infarction versus non-ST-elevation acute coronary syndrome treated with titanium-nitride-oxide-coated versus everolimus-eluting stents: insights from the BASE-ACS trial. *Minerva cardioangiologica* **2013**, *61*, 201-209.
28. Velders, M.A.; Boden, H.; van der Hoeven, B.L.; Liem, S.S.; Atary, J.Z.; van der Wall, E.E.; Jukema, J.W.; Schalij, M.J. Long-term outcome of second-generation everolimus-eluting stents and Endeavor zotarolimus-eluting stents in a prospective registry of ST-elevation myocardial infarction patients. *EuroIntervention : journal of EuroPCR in collaboration with the Working Group on Interventional Cardiology of the European Society of Cardiology* **2013**, *8*, 1199-1206, doi:10.4244/EIJV8I10A184.
29. De Luca, G.; Dirksen, M.; Spaulding, C.; Kelbæk, H.; Schalij, M.; Thuesen, L.; van der Hoeven, B.; Vink, M.; Kaiser, C.; Musto, C.; et al. Drug-eluting stents in patients with anterior STEMI undergoing primary angioplasty: A substudy of the DESERT cooperation. *Clinical Research in Cardiology* **2014**, *103*, 685-699, doi:10.1007/s00392-014-0702-6.
30. Huang, Y.; Ng, H.; Ng, X.; Subbu, V. Drug-eluting biostable and erodible stents. *Journal of Controlled Release* **2014**, *193*, 188-201, doi:10.1016/j.jconrel.2014.05.011.
31. Karjalainen, P.; Nammas, W.; Airaksinen, J. Optimal stent design: Past, present and future. *Interventional Cardiology* **2014**, *(2014) 6*, 29-44, doi:10.2217/ica.13.84.
32. Ribamar, C.; Almeida, B.; Costa, R.; Chamié, D.; Abizaid, A.; Perin, M.; Staico, R.; Feres, F.; Siqueira, D.; Veloso, M.; et al. Comparison of drug-eluting stents with durable or bioabsorbable polymer: Intracoronary ultrasound results of the BIOACTIVE trial ORIGINAL (NON-ENGLISH) TITLE Comparação de stents farmacológicos com polímero durável ou biorreabsorvível: Resultados do ultrassom intracoronário do estudo BIOACTIVE. *Revista Brasileira de Cardiologia Invasiva* **2014**, *22:3.*
33. Tuomainen, P.; Sia, J.; Nammas, W.; Niemelä, M.; Airaksinen, J.; Biancari, F.; Karjalainen, P. Pooled analysis of two randomized trials comparing titanium-nitride-oxide-coated stent versus drug-eluting stent in STEMI. *Revista espanola de cardiologia (English ed.)* **2014**, *67*, 531-537, doi:10.1016/j.rec.2014.01.024.

34. Bosiers, M.; Deloose, K.; Callaert, J.; Verbist, J.; Hendriks, J.; Lauwers, P.; Schroë, H.; Lansink, W.; Scheinert, D.; Schmidt, A.; et al. Superiority of stent-grafts for in-stent restenosis in the superficial femoral artery: twelve-month results from a multicenter randomized trial. *Journal of endovascular therapy* **2015**, *22*, 1-10, doi:10.1177/1526602814564385.
35. Chamié, D.; Almeida, B.O.; Grandi, F.; Filho, E.M.; Costa, J.R.; Costa, R.; Staico, R.; Siqueira, D.; Feres, F.; Tanajura, L.F.; et al. Vascular response after implantation of biolimus A9-eluting stent with bioabsorbable polymer and everolimus-eluting stents with durable polymer. Results of the optical coherence tomography analysis of the BIOACTIVE randomized trial ORIGINAL (NON-ENGLISH) TITLE Resposta vascular após implante de stents liberadores de biolimus A9 com polímero bioabsorvível e stents liberadores de everolimus com polímero durável. Resultados da análise de tomografia de coerência óptica do estudo randomizado BIOACTIVE. *Revista Brasileira de Cardiologia Invasiva* **2015**, *23*, 28-37, doi:10.1016/j.rbciev.2015.02.001.
36. Sia, J.; Nammas, W.; Niemelä, M.; Airaksinen, J.K.E.; Lalmand, J.; Laine, M.; Tedjokusumo, P.; Nyman, K.; Biancari, F.; Karjalainen, P.P. Gender-based analysis of randomized comparison of bioactive versus everolimus-eluting stents in acute coronary syndrome. *Journal of Cardiovascular Medicine* **2015**, *16*, 197-203, doi:10.2459/jcm.0000000000000086.
37. Das, M. Current status on drug-eluting devices in dialysis access. *CardioVascular and Interventional Radiology* **2016**, *39*, S58-S59, doi:10.1007/s00270-016-1405-3.
38. Karjalainen, P.P.; Niemelä, M.; Pietilä, M.; Sia, J.; de Belder, A.; Rivero-Crespo, F.; de Bruyne, B.; Nammas, W. 4-Year outcome of bioactive stents versus everolimus-eluting stents in acute coronary syndrome. *Scandinavian Cardiovascular Journal* **2016**, *50*, 218-223, doi:10.1080/14017431.2016.1177198.
39. Varho, V.; Kiviniemi, T.O.; Nammas, W.; Sia, J.; Romppanen, H.; Pietilä, M.; Airaksinen, J.K.; Mikkelsson, J.; Tuomainen, P.; Perälä, A.; et al. Early vascular healing after titanium-nitride-oxide-coated stent versus platinum-chromium everolimus-eluting stent implantation in patients with acute coronary syndrome. *The international journal of cardiovascular imaging* **2016**, *32*, 1031-1039, doi:10.1007/s10554-016-0871-7.
40. disclosed, N. ESC 2017: Hexacath's titanium-nitride-oxide coated stent matches Boston Scientific's Synergy for acute coronary syndrome. **2017**.
41. disclosed, N. Study Results: Titaniam-nitride-oxide (TiNO) Coated Stent is Non-inferior to Bioabsorbable Polymer Everolimus-eluting Stent (EES), for Patients Presenting with Acute Coronary Syndrome (ACS). **2017**.
42. Karjalainen, P.; Paana, T.; Ylitalo, A.; Sia, J.; Nammas, W. Optical coherence tomography follow-up 18 months after titanium-nitride-oxide-coated versus everolimus-eluting stent implantation in patients with acute coronary syndrome. *Acta radiologica (Stockholm, Sweden : 1987)* **2017**, *58*, 1077-1084, doi:10.1177/0284185116683573.
43. Karjalainen, P.P.; Nammas, W. Titanium-nitride-oxide-coated coronary stents: insights from the available evidence. *Annals of medicine* **2017**, *49*, 299-309, doi:10.1080/07853890.2016.1244353.
44. Karjalainen, P.P.; Niemelä, M.; Laine, M.; Airaksinen, J.K.E.; Ylitalo, A.; Nammas, W. Usefulness of Post-coronary Dilation to Prevent Recurrent Myocardial Infarction in Patients Treated With Percutaneous Coronary Intervention for Acute Coronary Syndrome (from the BASE ACS Trial). *Am. J. Cardiol.* **2017**, *119*, 345-350, doi:10.1016/j.amjcard.2016.09.057.
45. Nammas, W.; Airaksinen, J.K.E.; Romppanen, H.; Sia, J.; de Belder, A.; Karjalainen, P.P. Impact of Preexisting Vascular Disease on the Outcome of Patients With Acute Coronary Syndrome: Insights From the Comparison of Bioactive Stent to the Everolimus-Eluting Stent in Acute Coronary Syndrome Trial. *Angiology* **2017**, *68*, 513-518, doi:10.1177/0003319716664266.
46. Nammas, W.; de, B.A.; Niemelä, M.; Sia, J.; Romppanen, H.; Laine, M.; Karjalainen, P. Long-term clinical outcome of elderly patients with acute coronary syndrome treated with early percutaneous coronary intervention: insights from the BASE ACS randomized controlled trial:

bioactive versus everolimus-eluting stents in elderly patients. *European journal of internal medicine* **2017**, *37*, 43-48, doi:10.1016/j.ejim.2016.07.027.
47. Daoud, F.; Letinier, L.; Moore, N.; Coste, P.; Karjalainen, P. Efficacy and safety of percutaneous coronary interventions using titanium-nitride-oxide coated bioactive stents versus drug-eluting stents in coronary artery disease. A systematic literature review and meta-analysis. *Circulation* **2018**, *138 Supplement 1.*
48. de Winter, R.J.; Katagiri , Y.; Asano, T.; Milewski, K.P.; Lurz, P.; Buszman, P.; Jessurun, G.A.J.; Koch, K.T.; Troquay, R.P.T.; Hamer, B.J.B.; et al. A sirolimus-eluting bioabsorbable polymer-coated stent (MiStent) versus an everolimus-eluting durable polymer stent (Xience) after percutaneous coronary intervention (DESSOLVE III): a randomised, single-blind, multicentre, non-inferiority, phase 3 trial. *The Lancet* **2018**, *391*, 431-440, doi:10.1016/s0140-6736(17)33103-3.
49. Hsu, C.; Kwan, G.; Singh, D.; Rophael, J.; Anthony, C.; van Driel, M. Angioplasty versus stenting for infrapopliteal arterial lesions in chronic limb-threatening ischaemia. *Cochrane Database of Systematic Reviews* **2018**, doi:10.1002/14651858.CD009195.pub2.
50. Hernandez, J.M.D.; Moreno, R.; Gonzalo, N.; Rivera, R.; Linares, J.A.; Fernandez, G.V.; Menchero, A.G.; del Blanco, B.G.; Hernandez, F.; Gonzalez, T.B.; et al. The Pt-Cr everolimus-eluting stent with bioabsorbable polymer in the treatment of patients with acute coronary syndromes. Results from the SYNERGY ACS registry. *Cardiovasc. Revascularization Med.* **2019**, *20*, 705-710, doi:10.1016/j.carrev.2018.10.019.
51. Kayssi, A.; Al-Jundi, W.; Papia, G.; Kucey, D.; Forbes, T.; Rajan, D.; Neville, R.; Dueck, A. Drug-eluting balloon angioplasty versus uncoated balloon angioplasty for the treatment of in-stent restenosis of the femoropopliteal arteries. *Cochrane Database of Systematic Reviews* **2019**, doi:10.1002/14651858.CD012510.pub2.
52. Kuroda, K.; Otake, H.; Shinohara, M.; Kuroda, M.; Tsuda, S.; Toba, T.; Nagano, Y.; Toh, R.; Ishida, T.; Shinke, T.; et al. Effect of rosuvastatin and eicosapentaenoic acid on neoatherosclerosis: The LINK-IT Trial. *EuroIntervention : journal of EuroPCR in collaboration with the Working Group on Interventional Cardiology of the European Society of Cardiology* **2019**, *15*, E1099-E1106, doi:10.4244/eij-d-18-01073.
53. Nct. Rationale and Design of the Web basEd soCial Media tecHnology to Improvement in Adherence to Dual anTiplatelet Therapy Following Drug-Eluting Stent Implantation(WECHAT).
54. Kuno, T.; Takahashi, M.; Hamaya, R. The Randomized TIDES-ACS Trial. *JACC: Cardiovascular Interventions* **2020**, *13*, 2444, doi:10.1016/j.jcin.2020.08.004.
55. Lemkes, J.S.; Janssens, G.N.; Van Der Hoeven, N.W.; Jewbali, L.S.D.; Dubois, E.A.; Meuwissen, M.M.; Rijpstra, T.A.; Bosker, H.A.; Blans, M.J.; Bleeker, G.B.; et al. Coronary Angiography after Cardiac Arrest without ST Segment Elevation: One-Year Outcomes of the COACT Randomized Clinical Trial. *JAMA Cardiology* **2020**, *5*, 1358-1365, doi:10.1001/jamacardio.2020.3670.
56. Sun, G.; Lei, L.; Liu, L.; Liu, J.; He, Y.; Guo, Z.; Dai, X.; He, L.; Chen, S.; Liang, Y.; et al. Rationale and design of the Web-basEd soCial media tecHnology to improvement in Adherence to dual anTiplatelet Therapy following Drug-Eluting Stent Implantation (WECHAT): protocol for a randomised controlled study. *BMJ open* **2020**, *10*, e033017, doi:10.1136/bmjopen-2019-033017.
57. Wardle, B.; Ambler, G.; Radwan, R.; Hinchliffe, R.; Twine, C. Atherectomy for peripheral arterial disease. *Cochrane Database of Systematic Reviews* **2020**, doi:10.1002/14651858.CD006680.pub3.
58. Gómez-Lara, J.; Oyarzabal, L.; Brugaletta, S.; Salvatella, N.; Romaguera, R.; Roura, G.; Fuentes, L.; Pérez Fuentes, P.; Ortega-Paz, L.; Ferreiro, J.L.; et al. Coronary endothelial and microvascular function distal to polymer-free and endothelial cell-capturing drug-eluting stents. The randomized FUNCOMBO trial. *Revista espanola de cardiologia (English ed.)* **2021**, *74*, 1013-1022, doi:10.1016/j.rec.2021.01.007.
